# Medical Items and News of Interest to the Physician

**Published:** 1882-07

**Authors:** 


### Medical Items and News,

### For the Use of Physicians.

CULLED FROM THE STANDARD MEDICAL JOURNALS OF THE WORLD.

NOTE.—These formulæ are intended only for the regular practitioner of medicine, and should be employed only by him, or under his immediate supervision.

**Prurigo.**

℞

Phenate of soda, 25 grammes.
Glycerine, 100 grammes.
Cologne, 75 grammes.
Distilled water, 300 grammes.

M. With a syringe apply occasionally over the part affected.—*Union Medicale.*

**Menstrual Flow Scanty and the Liver Sluggish.**

℞

Podophylli resinæ, grs. vj;
Ext. hyoscyami, grs. xxiv;
Ext. nucis vomicæ, grs. iv;
Pil. aloes et myrrhæ, grs. xxx.

M. Divide into twelve pills, one to be taken at bedtime, three or four nights in succession.

**New Mode of Administering Castor Oil.**

To the oil add sufficient pulverized sugar, flavored with cinnamon or lemon, to convert the whole into a plastic dough; then form into boluses. Compound licorice powder may be substituted for the sugar. Children, says the *Boston Journal of Chemistry*, quarrel over this confection. For grown people the boluses may, in some instances, be enveloped in wafer paper.

**High Temperature.**

On the 17th of October, 1880, a soldier 26 years of age was admitted into the military hospital at Lour Palauka (Bulgaria), with intermittent fever. After the ague, the fever rose very high, as was apparent in heat of skin and frequency of pulse. At three o'clock in the afternoon the thermometer in the axilla indicated a temperature 41° C., equal to 106° F.; and later in the evening the temperature rose to 46° C., equal to 115° F. The respiration was 26 to the minute, and the pulse 115 to the minute. The skin was almost livid, and the eyes highly inflamed, with engorged blood vessels; vision and hearing regular, but the speech was tremulous. The temperature only remained at the highest point eight minutes, and fell to 97° F.—*Revista Madrid and Bulgarian Journal.*

**Psoriasis.**

℞

Acid pyrogallic, 5 to 18 grammes.
Suet or lard, 100 grammes.

M. Use 3 or 4 times a day for 3 or 4 weeks.

The pyrogallic acid produces a brown or black discoloration which disappears after some hours, but if too strong produces sometimes an erythema or redness, with ulceration. At the same time give internal remedies, particularly arsenical preparations.—*Union Medicale.*

**Mitral Affections.**

In the second period of mitral affections, when the tonic force of the cardiac muscle needs to be augmented to compensate for the disorders resulting from mitral contraction and from aortic feebleness, the two principal tonics which the heart needs are digitalis and the bromide of potassium. A maceration of digitalis in cold water for 12 hours, and the medium dose given the first day, lessening one tenth each day for six days, to prevent intolerance of the drug. During the next six days digitalis is not taken, and 15 to 30 grains of bromide of potassium are given daily in a solution of milk. After six days the medicines on alternate days, occasionally leaving a day of repose. giving no medicine. Digitalis must not be given when there is a granular fatness of the cardiac muscle.—Dujardin Beaumetz in *Union Medicale,*

**Therapeutical Indications for Ergot.**

Dr. E. Evetzky, in the *New York Medical Journal*, after a careful study of that drug, sums up his views regarding its action under five heads:

First: Disorders of the circulation and diseases of the organs of circulation. Second: Paretic conditions of the organs composed of organic muscular tissue, the circulatory system excepted. Third: Inflammatory and other morbid enlargements and growths. Fourth: Abnormal secretions. Fifth: Symptoms referable to the nervous system, and depending chiefly upon circulatory disorders within it. In regard to contraindictions to the use of ergot, it should be used with extreme caution in patients with an enfeebled heart. Pregnancy is not an absolute contra indication. The use of the drug should be suspended during menstruation, unless it is given for some special condition of that function. To avoid disturbing the digestion it is best to give the drug by the rectum or hypodermically.

**Hiccough.**

*Le Medicin Practicien* gives the following formula for hiccough, which it accredits to Dr. Park:

℞

Potassii bromidi, ʒ j.

Tr. sumbul, ʒ ss.

Tr. hyoscyami, ʒ j.

Aquæ camphoræ, q. s. ad., ℥ ij.

M. Sig. A tablespoonful every two hours.

**To Hasten the Action of Quinine.**

Dr. Starke (*Berliner Klin. Wochschrift*) advises that before swallowing powders or pills of quinia, a weak tartaric acid lemonade be taken. This procedure not only greatly accelerates the solution and absorption of the quinia, rendering its physiological action much more prompt, but also obviates that unpleasant gastric irritability so common after the administration of large doses of this drug.—*Medical Press.*

**Crupal Pneumonia.**

According to the author—in uncomplicated croupal pneumonia—iodine and iodine potassium are specified if given within 36 hours after the commencement of the attack. Of 98 cases treated, and 10 of them with details of the treatment, the result was very satisfactory. He considers that iodine destroys the diseased germs and aborts the disease rapidly. The following formulæ were used:

℞
Tr. iodine, 5 drops.
Distilled water, 120 grammes.

Dose—A large spoonful every hour. Iodide potassium 1½ grains in sweetened water every hour.—Dr. Schwarts, in *Revista Madrid.*

**Blind Piles.**

Dr. Blaschko, of Berlin, has, as stated in the *Wein. M. Bl.*, used with sure success ergotin for blind piles forced outside of the rectum. Mix ergotin with one hundred times its weight of distilled water, saturate with this some linen or muslin and apply it to the piles. The pain ceases and the lumps move back into the rectum. For the same purpose Dr. Posqua, of Italy, recommends the following:

Extract of belladonna, gr. v.
Iodoform, gr. j.
Acetate of lead, gr. j.
Vaseline, gr. 75.

Mix. Apply three or four times a day, after bathing the swelling with warm water.

**Coffee Hypodermically in Opium Poisoning.**

Dr. M. A. Pallen, N. Y., reports several cases of opium poisoning and morphia vomiting, which were treated chiefly by fluid extract of Java coffee, administered hypodermically in quantities of 15, 20 and 30 minims at a time. Recovery was quite rapid. It was observed that, if the injection was previously warmed, no abscesses were produced by it, but a cold injection was followed by inflammation or abscess—*Medical Record.*

**Oil of Turpentine in Mixtures.**

Oil of turpentine is a medicament that few patients can take without disgust. Ordinary sulphuric ether, luckily, has the property of modifying its persistently unpleasant flavor. The following mixture has been found to be very beneficial in vesical catarrh, neuralgia, and sciatica:

Oil of turpentine, ʒ ij.
Sulphuric ether, ʒ j.
Mix by shaking violently and add syrup of orange flowers, ℥ j.
Water, ℥ iv.

A teaspoonful to be taken every two hours.

**Nasal Catarrh.**

Dr. N. Falliott, writing to the *British Medical Journal*, states that coryza or nasal catarrh may be cured in a few hours if taken at the onset, or at most twelve hours afterwards, by the inhalation of a spray of sulphate quinine. The solution used is made by dissolving four grains of quinine in an ounce of water, with just enough of sufficient dilute sulphuric acid to dissolve it, and scenting with any agreeable perfume. The solution is injected up the nostrils in the form of spray with an ordinary hand-ball spray producer, in such a way that the quinine can be tasted at the back of the mouth. This is done at ever hour or oftener, according to the urgency of the symptoms. He states that this remedy has been tried with success in hay fever, and that if nasal catarrh is of parasitic origin, as he stongly suspects, the action of quinine (as an antiseptic ?) is at once apparent. It might be added that, even supposing catarrh to be the result of sudden change of temperature, the action of quinine in contracting the superficial capillaries would be quite as obvious. It is somewhat surprising that this property of quinine does not appear to have been tried for chilblains in the itching state, when the capillary vessels are dilated.—*New Remedies.*

**Pneumonia.**

Dr. S. W. Francis, Newport, Rhode Island, (*Medical Record*) claims that by the use of inhalations of sulphuric ether he has had very good results in acute pneumonia. He claims that "if seen early during the first stage, the inhalation of sulphuric ether, for thirty minutes every six hours, would arrest many severe and protracted cases." Similar inhalations have been before proposed by Dr. Francis in bronchitis. That such inhalations might have some influence on pneumonia there can be no doubt, but Dr. Francis' claim is a little too positive.

---

**Diabetes.**

Professor Da Costa reports six cases of diabetes insipidus, treated with ergot, with five recoveries. The remedy is given in the form of fluid extract and not less than three fluid drachms per day, and this amount should be gradually increased to double its quantity. It may be well to follow the ergot, after the amount of urine has been reduced to the normal, with strychnia. In other cases where the ergot must be continued for some time, cod liver oil is a good adjunct.—*Medical News.*

---

**Iron Preparations.**

Dr. Alfred W. Perry writes in the *Western Lancet:* In cases of debility, prostration, or loss of appetite, preparations of iron, alone or variously combined with bitter tonics, are seemingly indicated clearly, and are very generally used. But in many cases they do harm, either from their being administered at a wrong time or because they are not tolerated under any form or circumstance. The greatest abuse of iron is where it is given for loss of appetite or difficult indigestion, and when it is given within half an hour before eating or within three hours after. We have found entirely to our own satisfaction, both by clinical observation and by experiment, that iron preparations introduced into the stomach while digestion is going on, either hinder or arrest the process.

---

**Hydrate of Chloral for Preserving Urine.**

Dr. F. C. Curtis, in the *Medical Annals*, recommends hydrate of chloral for preserving urine. In August last he received a specimen of urine, four ounces, containing five grains of chloral to the ounce. No special care was taken to facilitate its preservation. It has been simply corked, and has been several times opened. It is now perfectly transparent, of a clear amber color, and has a fresh, urinous, slightly balsamic odor. A slight semi-flocculent deposit covers the bottom of the vial. The chemical analysis is identical with the notes made when it was recent: sp. gr. 1.015, acid, albumen one-fourth. The preservation of the albumen is a marked test. On microscopic examination, the epithelial cells are as perfect as in recent urine. No casts were noted and none are now seen. The specimen seems to be quite the same as when freshly voided, and is manifestly instructive and of considerable interest.

---

**Infantile Convulsions.**

Dr. Windelschmield writes that iodoform is very efficacious in convulsions of infants and that its action is not purely palliative but often radical. He uses the following formula:

℞

Iodoform 10 to 50 centigrammes.
Iodide of potassium, 1 gramme.
Wine of tokay, 10 grammes.

M. Take in three doses.—*Journal de Therapeutique.*

---

**The Anæsthetic Mixture at St. Bartholomew's Hospital.**

At St. Bartholomew's, adult patients are not chloroformed, but are anæsthetized with a mixture of ether and nitrous oxide. It is claimed that the mixture works more quickly and with less discomfort than pure ether. It is given with an inhaler, which consists of a mouthpiece connected with a rubber tube with a reservoir containing the anæsthetic, and arranged so that any desired amount of air can be admitted. Chloroform is given to patients under fourteen years of age.—*Boston Medical and Surgical Journal.*

---

**Sore Nipples.**

Dr. Favre (*Petersburg, Med. Woch.*) distinguishes two kinds of this lesion—fissures and erosions—and, believing that the latter are much induced by the modern tight-fitting dresses and the pressure of the corset, warns pregnant women against this mode of procedure. As a means of treatment he recommends the sprinkling of the sores with bismuth, or employing this as an ointment, in the proportion of two drachms to half an ounce of vaseline. In some cases twenty-four hours application of this means has removed all suffering, and allowed suckling to be resumed.—*Med. Times and Gaz.*

**Erysipelas.**

℞
Acid phenic,
Alcohol, aa 1 part,
Spts. turpentine, 2 parts,
Tr. iodine, 1 part.
Glycerine, 5 parts.

This mixture produces no pain, and can be applied every two hours.

Internally, quinine and digitalis when there is fever, vomiting, etc.—Dr. Rothe in *Memorabilien*.

---

**Antiseptic Powder for Wounds.**

MM. Bruns and Kersh give the following in the *Union Pharmaceutique:*

Powdered resin, 60 parts.
Stearic acid, 15 parts.

Melt at a gentle heat, and add when nearly cool 25 parts of crystallized carbolic acid, thoroughly incorporating the ingredients. When quite cold powder, and mix with 700 parts of precipitated chalk. The powder is to be sprinkled on the wound from a box provided with a perforated cap, and a cover to keep the contents from the air.

---

**Rheumatism.**

Dr. James H. Wilkes, of Columbia, Tenn., writes to the *Ther. Gazette*, claiming to have cured cases of articular rheumatism that had resisted the usual remedies, including salicylic acid, with *manaca.* This is one of the new remedies brought to notice by Park, Davis & Co. He gave five drops of the fluid extract of manaca every four hours. Next day the dose was increased to eight drops every four hours, with the result of enabling the patient to rise from his bed on the third day and walk out.

---

**A Simple Aperient.**

Dr. Weir Mitchell, in a case of nervous affection in which constipation existed, once recommended the patient to take each morning, on rising, a tumblerful of water in which was dissolved a teaspoonful of common salt. The water should be cold, or it might nauseate. "This simple aperient I frequently employ in cases of constipation, and generally find it efficient. There is great advantage in starting the bowels and in keeping them in a soluble condition, particularly in cases of nervous disorder in women, as it sometimes clears up obscure points in the case, and at all events eliminates one source of error."

**Diarrhœa.**

Prof. Wm. Thompson, of the University of the City of New York, recommends the following as a remedy for diarrhœa:

℞
Plumbi acetatis, gr. xvi.
Pulv. camphoræ, gr. xij.
Pulv. opii., gr. iij.
Bismuth. subcarb. gr. xij.
Ext. gentian, q. s.

Make into 12 pills. Dose, one pill every hour to three hours, according to severity of disease.

---

**Syphilis.**

DR. JOHN V. SHOEMAKER, of Philadelphia, has treated 103 cases of syphilis by hypodermic injections. In these 103 cases he gave 2,018 injections in the course of 161 days. He injected a solution containing 6 grains of hydrarg. bichlor. to the ounce of water. He commenced generally with one-eighth of a grain, that is ten minims of the above solution. Thirty-five patients failed to continue treatment; sixty-eight took from day to day injections until they were discharged, and of this number forty-one were entirely cured; the remainder, twenty-seven, had relapses at various times.—*Medical Bulletin.*

---

**Muriate Tincture of Iron Pomade.**

Dr. Casurini has used the perchloride of iron in many diseases of the scalp. He uses a pomade of 1, 2 or 3 grammes with 30 grammes of suet, or as a lotion with water. The affections he has tried it in are psoriasis, acute and chronic, lichen, eczema—which were cured.

1. The perchloride of iron is the most efficacious remedy against purpura hemorrhagica and purpura simplex.

2. It is very useful in all chloranemic conditions of the system which accompany diseases of the scalp, like rupia, ecthyma and impetigo.

3. It exercises a beneficial effect very promptly on ulcers, either scrofulous or syphilitic.

4. It also acts well in squamous diseases of the scalp.

---

**Sedative Emmenagogue.**

For a day or two antecedent to the actual commencement of the catamenial flux, not infrequently women suffer acute pain in the pelvic region, doubtless due to hyperæmia and hyperæsthesia of the reproductive belongings. To obviate this I have found no treatment give such satisfactory results as the following:

℞
Codeæ sulphatis, gr. j.
Chloral hydratis.
Ammonia bromidi, āā gr. xx.
Aquæ camphoræ, ℥ j.

M. Ad unum haustum. S. Take at bedtime.

A repetition of the dose at *that* period is rarely necessary. In some cases a warm sitz-bath of fifteen minutes duration before retiring, is a valuable adjunct.

**Baths for the Newly Born.**

Dr. F. Winckle, of Dresden, makes the novel suggestion of keeping certain newly-born children permanently in warm water. This he considers more useful than rolling them in cotton wool, applying warm bottles, and keeping them in warm rooms. The following abnormal conditions are mentioned as being suitable for the permanent bath: 1. Children born between the 28th and 36th weeks. 2. Children born asphyxiated and weak from flooding during labor, or who have accidentally lost blood from the stump of the cord. 3. Where there is disease or fretting of the skin. 4. In emaciation, to prevent bed sores. The author has employed this treatment successfully in cases such as those above mentioned, and gives details of temperatures and results.—*Glasgow Medical Journal.*

**Cholera Infantum.**

Dr. Boing, of Germany, has treated this disease with the happiest results. His method consists in the use of quinine in fractional doses, in the reduction of milk with half water, a little warm, in large doses of wine and of ether. The number of children treated was fifty, and the ages varying from two months to four years, the children of a greater age not being of the number. Children under one year of age were confined to breast milk, the others with artificial food. In the treatment of the poor, for wine there was substituted the ether which was acidified. For children of 5 to 10 months, ½ to 1 grain quinine was given from hour to hour. When the passages and vomitings were acid phosphate of lime was added, and continued as long as the urine indicated acidity. For a drink, gum Arabic water mixed with Manzarutta water. Wine was given by the spoonful every quarter of an hour to an hour. When milk is not borne, the white of an egg mixed with gum Arabic, can be substitued given warm and slightly sweetened with sugar.

In no place have I employed astringents, opiates, or cold or cutaneous revulsives. I have abandoned phenic acid, creosote, and also calomel, which I consider worse than opiates, and cold applications. What decided me to aban don what is called the rational treatment, was the great mortality of 50, 70, or 90 in 100 cases, which shows that something else ought to be adopted.—*Revista Medico Buenas Ayres.*

**Blennorrhagia.**

The Paris *Medical* reports that Zeitlin has treated fourteen cases of blennorrhagia with chlorate of potash, administered internally, in daily doses of three grams (grs. xl) according to Dachman's method. The results have always been satisfactory. After a few days micturition becomes painless, erections cease, and the discharge is less abundant, and more serous.

The happy effects of this salt are due to the rapidity with which it is excreted by the kidneys, without any change in its composition, and to its local action on the urethral mucous membrane.

Good results have also been obtained with chlorate of potash, prescribed as an injection, and in cases of blennorrhagic or other cystitis its internal use has been found beneficial.—*Med. and Surg. Reporter.*

**A New Method of Embalming Bodies and Preserving Tissues.**

Dr. Virodtzeff recommends the following preparation as an efficient agent in the embalming of bodies and the preservation of tissues:

Thymol, 5 parts.
Alcohol, 45 parts.
Glycerine, 2.160 parts.
Water, 1.080 parts.

It is cheap, innocuous, free from unpleasant odor, possesses the property of keeping the body soft, elastic, fresh, and life-like, and does not ruin instruments. Thymol is selected as being superior to other antiseptics, and glycerine is added, both on account of its own preservative qualities, and to retard the evaporation of the fluid. For the preparation of tissues the same solution is used. If the cadaver be quite lean, or the tissues very delicate, equal parts of water and glycerine (1.620 of each) are combined with the above quantities of thymol and alcohol. To inject a body, half its weight of the fluid is necessary. A properly embalmed cadaver may be preserved indefinitely under ordinary circumstances, gradually shrinking and mummifying without putrefaction. Specimens are either to be injected with or macerated in this fluid. Maceration must not be too prolonged—the appearance of the specimen should act as a guide. The part, after having been thoroughly cleansed in water, and prepared, may then be exposed for months to the air without losing its consistence, form, and color. Permanent specimens may be inclosed in a hermetically-sealed glass vessel containing a little of the same solution. The *Medical Record* says that Dr. Peabody has used this preserving fluid with excellent result in the New York Hospital museum.

**Constipation.**

Where constipation is due to torpor of the muscular layer of the intestine, combined with defective secretion of the mucous membrane, Dr. Bartholow uses either one of these formulæ:

℞
Tc. nucis vomicæ.
Tc. belladonæ.
Tc. physostigmæ, āā f. ʒ ij. M.

SIG.—Thirty drops in water, morning and evening, or

℞
Ex. physostigmæ.
Ex. belladonæ.
Ex. nucis vomicæ, āā gr. v.
M. et. ft. in. pil. No. x.

SIG.—One pill at bedtime.

—*Clinical News.*

**Acute Rheumatism.**

In the *British Medical Journal*, Dr. P. W. Latham, asks: "Is salicylic acid a specific for acute rheumatism?" and gives a short summary of his theory of the pathology of acute rheumatism.

1. A nervous center exists, which controls the nutrition of the muscular and other tissues, and which has been termed the "inhibitory chemical center."

2. The action of cold, lowering the power of this center, may modify the nutrition of the tissues and lead to excessive formation of lactic acid and other products.

3. The presence of lactic acid, in abnormal amount, produces functional changes in these nerve-centers, and develops symptoms of acute rheumatism similar to the production of symptoms in locomotor ataxy, with its joint-affections, by organic changes.

4. If portions of the vagus be involved, then cardic, pulmonary, or pleuritic complications may develop.

5 Salicylic acid combines with the antecedents of lactic acid, and so prevents its formation.

6. If the remedy be discontinued too early, a relapse will certainly take place. As much of the remedy should be given from the first as can be borne, and great care must be taken to secure a pure form of the salicylic acid; that which is artificially prepared is often dangerous to administer, while pure acid seldom causes unpleasant symptoms.—*London Medical Record.*

**Hydrophobia Treated with Pilocarpine.**

To prevent the multiplied trials of useless drugs, it is well to note the unsuccessful use of remedies in hydrophobia. A case in Paris has been reported in *La France Medicale*, in which pilocarpine was employed without apparently retarding for a moment the fatal result. The patient was a man, aged thirty-eight, who was bitten on the 23d of February by a rabid dog. The wound, which was situated on the hand, was cauterized by carbolic acid, and cicatrized slowly. For two months no symptoms appeared. Then a dull pain seemed to start from the wound and to ascend the arm to the shoulder and trunk. Two days later the first paroxysms occurred, and the disease ran a rapid course, with its customary symptoms. Six injections of pilocarpine were given, two centigrammes in each, and caused abundant salivation and less copious diaphoresis. When the effect of each was over, the patient experienced a notable relief to his sufferings; but the course of the malady appeared to be altogether uninfluenced. The salivation which was produced by the later injections was attended by terrible paroxysms of suffocation, followed, however, by distinct relief. The loss of liquid occasioned by the treatment increased the thirst; but deglutition was impossible. A tube was therefore passed down the œsophagus, and as much liquid as possible introduced into the stomach. The operation was well borne, and, on repetition, the patient himself introduced the tube. Forty-eight hours after the commencement of the treatment the patient died suddenly. He was sitting on his bed, talking, when his head sank forward on his chest, he gave one or two inspirations, and was dead. Sudden death from cardiac failure is too common in hydrophobia for the result to be ascribed with certainty to the pilocarpine, but it should teach caution in the use of so depressing an agent in the disease.

---

**Quebracho Bark in Dyspnœa.**

Some time since, Dr. Penzoldt published in *Berlin Kin. Wochen.* a very interesting paper on quebracho, in which he stated that he had obtained most striking and unexpected effects from that drug in different forms of dyspnæa, but that as a febrifuge it had failed in his hands. Dr. Berthold, of Dresden, has since then experimented with the remedy in fifteen cases of intense dyspnœa, with the following results: In a severe case of asthma convulsorum a teaspoonful of the tincture of quebracho gave speedy relief, and three doses at hourly intervals effected a complete cure. In a case of emphysema with asthma a teaspoonful of the tincture was administered every three hours; the dyspnœa did not yield until the third day, but the asthmatic attack was decidedly shortened by the remedy. In a case of emphysema complicated with pulmonary catarrh and pregnancy, the respiration fell in one day from forty-eight to thirty-two under the use of quebracho. In a case of pleurisy, and one of chronic pulmonary catarrh, the remedy caused no improvement at all in the dyspnœa. In a case of mitral insufficiency and stenosis, and in two cases of fatty heart, it exerted a very decided palliative action on the dyspnœa. Of six cases of phthisis with great dyspnœa, only two obtained any relief from the quebracho, but in these two cases the relief was very marked. In one of these cases of phthisis the diarrhœa stopped after the first dose of the remedy. Dr. Berthold has found the alcoholic extract of quebracho, that is, the resinous residue which remains after digestion, and is of course only soluble in alcohol, to be a very excellent remedy for diarrhœa. He administered it in five cases of acute and chronic intestinal catarrh with very prompt effect.

Dr. Picot, of Carlsruhe, writes that he tried the tincture of quebracho in three cases of dyspnœa, due respectively to catarrhal pneumonia, bronchial asthma and valvular lesion, with very satisfactory effects. He also used it himself while doing some mountain climbing on his vacation, and found that it enabled him to climb with much greater ease and comfort—for instance, a certain expedition caused the pulse to rise from sixty-four to ninety-four, and the respiration from sixteen to forty-two, with a very unpleasant sensation of shortness of breath, but after a dose of half an ounce of the tincture, the same ascent only caused the pulse to rise to eighty and the respiration to thirty. A corpulent man and a nervous woman, who suffered greatly from dyspnœa after moderately rapid walking on level ground, were able to take the same amount of exertion without distress after a dose of from two and a half to four drachms of quebracho.

In *El Genio Medico-Quirurgico, Madrid*, Dr. Fr. Simon, of Nieto, publishes an interesting report on the above substance, from which we extract as follows:

Quebracho is the bark of a tree (aspidosperma quebracho blanco) which grows in South America; in color it resembles calisaya bark, and has a hot, bitter, and intensely disagreeable, acrid taste. Penzoldt first noticed its medicinal value; others, among whom Berthold, Krauth, Schutz and Laquer, have also experimented with it. All the observations thus recorded agree with those made by Dr. Simon, and point to quebracho as a specific in dyspnœa, whether resulting from asthma, or chronic or acute affections of the respiratory or circulatory systems, or when due to emphysema and pulmonary catarrh.

Two methods of preparing quebracho have been recommended by Penzoldt; the one, a tincture (1 to 10), of which the dose is from forty-five minims to one drachm, daily; the other an aqueous extract, which is obtained in the following way: 150 grains pulverized quebracho are digested in three ounces of alcohol; they are then evaporated and dissolved in water; the solution is filtered and evaporated to dryness. This forms the dry extract, of which the dose is from 15 to 30 grains daily; or it is dissolved in twenty times its weight of boiling water, and then constitutes the liquid extract, which Penzoldt prefers to any other form, not excluding aspidospermine (an alkaloid obtained by Fraude); two to four teaspoonfuls of the liquid extract may be given daily.

Dr. Simon details six different cases in which he administered quebracho successfully, besides some interesting experiments made upon himself, and he recommends the two following formulæ:

℞

Tr. quebracho (1 to 10), ʒ j

Water, f ℥ iiss

Syrup, f ℥ ss.

M. Sig.—A tablespoonful every four hours.

℞

Dry ext. quebracho, gr. xxx
Water, f ℥ jss
Syrup, f ℥ j.

M. Sig. A teaspoonful every half hour, until relief is obtained.

He concludes his report with the following observations:

1. Quebracho is a substance possessing the property of moderating respiratory movements; it acts, in truth, as the digitalis of the lungs.

2. It relieves dyspnœa, whether resulting from purely nervous disorders, or when due to anatomical alterations in the respiratory or circulatory apparatus.

3. Its action is immediate and its effects safe, at least so far as observed.

4. Its effects on dyspnœa caused by circulatory troubles warrant the belief that it not only acts directly on the nervous system governing the respiratory movements, but that its influence extends over cardiac innervation.

5. Finally, it is believed that quebracho facilitates expectoration.—*Medical and Surgical Reporter.*

---

**Epilepsy.**

M. Ball, the present professor of mental diseases at the Paris School of Medicine, considers that the drugs most used in epilepsy prove much more efficacious when taken in combination with each other, than when one of them is administered singly. The alkaline bromides, particularly the bromide of ammonium, with belladonna and oxide of zinc, form the basis of treatment.

The following solution may be given in tablespoonful doses:

℞

Ammonii bromid., ʒ iiss.

Sodii bromid., ʒ iiss.

Aquæ destil., 1 oz. x. M.

At the commencement of treatment four tablespoonfuls of this solution may be taken during the day, and the dose increased to eight or ten tablespoonfuls if no appreciable effect is noticed after a few days.

Belladonna and oxide of zinc are administered in pill form, as follows:

℞

Ext. belladonnæ, gr. xv.

Zinci oxid, gr. xv.

Ft. pil. xl. M.

Sig.—One pill may be taken in the morning, another in the evening, at first; then the dose may be increased to four pills per diem.

If any degree of plethora exists the drastic purgatives should be resorted to, and in some cases benefit is obtained.

What is of importance to notice is the immediate beneficial effect of this combined treatment; this is sometimes remarked on the second day.

This method, like all other forms of treatment of epilepsy, should be continued for a long period, and should not be suddenly stopped; the doses should be progressively and slowly dimished when it is considered safe to lay aside the treatment.—*Med. & Surg. Reporter.*

---

**Scrofula and Tuberculosis.**

M. Grancher recently made to the Societe Medicale des Hopitaux of Paris an interesting communication on the above subject. The following are his general conclusions: 1. Tubercle is a fibro-caseous neoplasm, the development of which takes place in successive stages, during a longer or shorter period; this complete evolution may be accomplished in a few months, or it may last throughout the whole of life. It may, however, be arrested during the earlier stages, and never get beyond them. 2. Pathological anatomy and experimental pathology are to-day agreed to include under the term tuberculosis the greater number of affecions called scrofulous, as *local* tuberculoses. 3. Lupus and superficial inflamations of skin and mucous membranes, the last resort of those persist in regarding scrofula and tuberculosis as distinct, will probably be included in the same order in due process of time. 4. The necessities of practical medicine, which, after all, must first be reckoned with, do not permit all tubercular affections to be confounded together; on this account it is convenient to retain the word "scrofula" for those tubercular affections which are very slight and generally curable.—*Med. Times and Gaz.*

